# Interferon-β Induced *microRNA-129-5p* Down-Regulates HPV-18 E6 and E7 Viral Gene Expression by Targeting SP1 in Cervical Cancer Cells

**DOI:** 10.1371/journal.pone.0081366

**Published:** 2013-12-16

**Authors:** Jiarong Zhang, Shuangdi Li, Qin Yan, Xiaoyue Chen, Yixia Yang, Xuelian Liu, Xiaoping Wan

**Affiliations:** Shanghai First People's Hospital Affiliated to Shanghai Jiao Tong University, Shanghai, China; The University of Tennessee Health Science Center, United States of America

## Abstract

Infection by human papillomavirus (HPV) can cause cervical intraepithelial neoplasia (CIN) and cancer. Down-regulation of E6 and E7 expression may be responsible for the positive clinical outcomes observed with IFN treatment, but the molecular basis has not been well determined. As miRNAs play an important role in HPV induced cervical carcinogenesis, we hypothesize that IFN-β can regulate the expressions of specific miRNAs in cervical cancer cells, and that these miRNAs can mediate E6 and E7 expression, thus modulate their oncogenic potential. In this study, we found that miR-129-5p to be a candidate IFN-β inducible miRNA. MiR-129-5p levels gradually decrease with the development of cervical intraepithelial lesions. Manipulation of miR-129-5p expression in Hela cells modulates HPV-18 E6 and E7 viral gene expression. Exogenous miR-129-5p inhibits cell proliferation in Hela cells, promotes apoptosis and blocks cell cycle progression in Hela cells. SP1 is a direct target of miR-129-5p in Hela cells. This study is the first report of a cellular miRNA with anti-HPV activity and provides new insights into regulatory mechanisms between the HPV and the IFN system in host cells at the miRNA level.

## Introduction

Cervical carcinoma and cervical intraepithelial neoplasia (CIN) are caused by persistent infection with high-risk human papillomavirus (HPV) serotypes, most commonly HPV16 and HPV18 [1]. Therefore, treatment of HPV infection is the key to preventing cervical cancer and CIN. Two oncoproteins, E6 and E7, which encoded by HPV16 and HPV18, can bind to and stimulate the degradation of the tumor suppressors p53 and pRb [2]–[5]. IFN has been widely used in the treatment of CIN and cervical cancer. Down-regulation of E6 and E7 expression may be responsible for the positive clinical outcomes observed with IFN treatment, but the molecular basis has not been well determined.

MicroRNAs (miRNAs) are 19–25 nt regulatory RNAs that participate in the regulation of various biological functions as well as in defense against pathogens in numerous eukaryotic lineages. They are generally believed to act by binding to imperfectly complementary sequences in the 3′untranslated (UTR) region of the target genes, resulting in decreased translation or degradation of the target transcript. In particular, the sequence complementarily in the 6–8 base pair “seed region” at the 5′ end of the miRNA-mRNA heteroduplex seems to determine the specificity of miRNA-targetRNA interactions. MiRNAs can have pleiotropic effects on cell proliferation, apoptosis and cell differentiation [6]. Alterations in cellular miRNA patterns in cervical cancer tissue or cervical cancer cells have been reported. Downregulation of human miR-218 [7], [8] and miR-34a [9] in cervical cancer cells were addressed to the HPV 16 E6 oncogene, while inhibition of miR-21 in HPV 18-containing Hela cervical cancer cells causes a strong suppression of cell proliferation [10]. It thus seems that miRNAs play an important role in cervical carcinogenesis by HPV.

MiRNAs may play a role in IFN-β induced E6 and E7 repression. It has been 1shown miRNAs can be induced by IFN-β. In RSa cells, IFN-β can induce miR-431 expression, which may down-regulate IGF1R and IRS2 expression and consequently inhibit cell proliferation by suppressing the MAPK pathway [11]. In hepatocytes, IFN-β mediates modulation of the expression of numerous cellular miRNAs with nearly perfect complementarity in their seed sequences with the HCV RNA genome that are capable of inhibiting HCV replication and infection [12]. As miRNAs play an important role in HPV induced cervical carcinogenesis, we hypothesize that IFN-β can regulate the expressions of specific miRNAs in cervical cancer cells, and that these miRNAs can mediate E6 and E7 expression, thus modulate their oncogenic potential. To verify this, we screened for miRNAs expressed and differentially regulated in HPV-18 positive Hela cells following exposure to IFN-β, and we found that miR-129-5p to be a candidate IFN-β inducible miRNA which can downregulate E6 and E7 expression.

## Materials and Methods

### Cell lines and human tissues

The HPV 18-positive Hela cell line [13], HPV 16-negative Siha cell line [13], HPV-negative C33A cervical cancer cell line [13] and well-differentiated cervical squamous cell carcinoma HCC94 cell line [14] were used in this study. All of these cells were grown in Dulbecco's modified Eagle's medium (DMEM) with 10% FBS at 37°C and 5% CO_2_. The cell lines were harvested after IFN-β (3× 10^5^IU L^−1^) treatment for 2 h.

Normal cervix and cervical cancer tissues were obtained from women ranging in age from 28 to 77 years old. Four normal cervical samples were obtained from patients who underwent hysterectomy to treat other types of diseases such as myoma or adenomyosis. None of the patients had undergone hormone therapy, radiotherapy or chemotherapy before surgery. The stages and histological grades of these tumors were established according to the criteria of the International Federation of Gynecology and Obstetrics (FIGO). Each tissue sample was immediately snap-frozen in liquid nitrogen and stored at −80°C until RNA extraction. Prior written and informed consent was obtained from each patient, and the study was approved by the ethics committee of the Medical Faculty of Shanghai Jiao Tong University. Clinical and pathological data relating to the clinical samples are presented in Table 1.

**Table 1:** Clinicopathological parameters of endometrial cancer samples.

Characteristics	No. of patients(%)
Total	33 (100)
Age (years)	
≤50	22 (66)
>50	11 (34)
Histological type	
CIN I-II	6 (18.18)
CIN III	13 (39.39)
Ib2	9 (27.27)
IIa	5 (15.15)
Human papillomavirus	33 (100)

### Microarray expression profiling and data analysis

Total RNA in cells or tissues were isolated using Tri-reagent (Molecular Research Center, Cincinnati, OH) according to the manufacturer's instructions. After passing RNA quality measurements in a Nanodrop instrument, the samples were labeled using the miRCURY™ Hy3™/Hy5™ Power labeling kit and hybridized on the miRCURY™ LNA Array (v.11.0). The samples were hybridized on a hybridization station following the protocol outlined by the manufacturer. Scanning was performed with the Axon GenePix 4000B microarray scanner. GenePix pro V6.0 was used to read the raw intensity of the image. The threshold values used to screen differentially expressed miRNAs were fold changes ≥1.5 or ≤0.7, and *P* values ≤0.05 in t-tests were considered significant.

### Crucial seed sequence complementarity analysis

Array data processing and crucial seed sequence complementarity analysis were performed using the web sites (http://www.mirbase.org/, http://www.ncbi.nlm.nih.gov/sites/entrez?db=pubmed and http://www.ebi.ac.uk/Tools/emboss/align/).

### Analysis of miRNA and mRNA by qPCR

Total RNA was extracted from cultured cells and tissues using Tri-reagent. For miRNA analysis, mature miRNAs were reverse transcribed from total RNA using specific miRNA RT primers in the TaqMan MicroRNA Assays and reagents in the TaqMan MicroRNA Reverse Transcription kit. qPCR was performed using TaqMan MicroRNA Assay primers with the TaqMan Universal PCR Master Mix and analyzed with an ABI Prism 7000 Sequence Detection System (Applied Biosystems, Foster City, CA) according to the manufacturer's instructions. *U6B* was detected as an internal control to normalize all data. The assay names for *miR-129-5p* and *U6B* were hsa-mir-129-5p and RNU6B, respectively (Applied Biosystems). The cDNA was generated using the Prime Script RT Reagent Kit (TaKaRa, Dalian, PRC). For quantification of HPV18 *E6*, HPV18 *E7* and *ISG54*, real-time PCR was carried out on the ABI Prism 7000 Sequence Detection System with SYBR Premix Ex Taq (TaKaRa). The primer sequences are shown in Table 2. For all qPCR assays, expressions were calculated by the formula 2^(−ΔΔCt)^, where ΔCt is the difference between gene Ct and normalizer gene Ct. Ct represents the threshold cycle at which fluorescence rises statistically significantly above the baseline. Experiments were performed in triplicate in three independent experiments.

**Table 2:** Primer sequences for real-time PCR.

mRNA	Size (bp)	Primer sequence
ISG54	144	forward 5′–TTCAGCATTTATTGGTGGCAGAAG-3′
		reverse 5′- AACTCTAAAATCCCACCCTGCC-3′
ISG56	131	forward 5′- GTCGTCTCTGCGCATCTTGTT-3′
		reverse 5′- TGCAGATAGAAAGCCTGCCTG-3′
β-ACTIN	251	forward 5′- CAGCCATGTACGTTGCTATCCAGG-3′
		reverse 5′- AGGTCCAGACGCAGGATGGCATG-3′
HPV-18 E6		forward 5′- ATCCAACACGGCGACCCTACAA-3′
		reverse 5′- CTGGATTCAACGGTTTCTGG-3′
HPV-18 E7		forward 5′- ACCTTCTATGTCACGAGCAAT-3′
		reverse 5′- CGGACACACAAAGGACAGGGT-3′

### Cell transfections

Cells were washed with PBS and switched to antibiotic-free growth medium for 24–48 h before transfection. Cells were transiently transfected with *pre-miR-129-5p*, non-specific miRNA control (pre-miR-negative) or blank control (Applied Biosystems) in Opti-MEM (Invitrogen, Carlsbad, CA) using siPORT NeoFX Transfection agent (Applied Biosystems) following the manufacturer's protocol. Medium was replaced 8 h later. Equal copy numbers of plasmids were transfected using Lipofectamine 2000 (Invitrogen) following the manufacturer's protocol. Luciferase reporter vector bearing the target sites for *miR-129-5p* of the *SP1* 3′-UTR region was purchased by Shanghai choseasy co., Ltd. The detailed information on the constructs used in this experiment is shown in Table 3.

**Table 3:** Sequences of inserted fragments in the luciferase constructs.

Name	Sequence
Sp1	5′tcgagTGAAATACTTTTTAACAAAAAACAGATTCTATTGAAATACTTTTTAACAAAAAACAGATTCTATgc-3′
	5′ggccgcATAGAATCTGTTTTTTGTTAAAAAGTATTTCAATAGAATCTGTTTTTTGTTAAAAAGTATTTCAc-3′
Mutant SP1 (negative construct)	5′tcgagTGAAATACTTTTTAAGtttttACAGATTCTATTGAAATACTTTTTAAGtttttACAGATTCTATgc-3′
	5′ggccgcATAGAATCTGTAAAAACTTAAAAAGTATTTCAATAGAATCTGTAAAAACTTAAAAAGTATTTCAc-3′

### Proliferation assays

Cells (3000 per well) were plated in 96-well plates and grown for 72 h after transfection (final miRNA concentration of 100 nM) in DMEM/F12 containing 10% FBS. Cell proliferation was documented every 24 h for 3 days using the colorimetric MTT assay (Sigma, St. Louis, MO), and absorbance at 490 nm was evaluated by a Spectra Max 190 microplate reader (Molecular Devices, Sunnyvale, CA).

Cells, including transfected cells, were starved for 24 h by replacing the medium with phenol red-free and serum-free DMEM/F12 medium (HyClone Laboratories, Logan, UT), and appropriate vehicle control with phenol red–free medium containing 5% charcoal-stripped FBS (HyClone Laboratories) for 48 h. Cell proliferation was documented every 24 h for 2 days. Within an individual experiment, proliferation under each condition was tested in triplicate, and the overall experiment was repeated at least twice.

### Cell cycle analysis

Cells were fixed with 70% ethanol at 72 h after transfection and stained with 25 µg/mL PI (Roche Molecular Biochemicals, Indianapolis, IN) in FACS buffer (PBS containing 0.1% bovine serum albumin (BSA), 0.05% of Triton X-100, and 50 µg/mL of RNase A). After 30 min at room temperature protected from light, the cells were analyzed via flow cytometry using a Becton Dickinson FACScan, and the fractions of the cells in the G0/G1, S and G2/M phases were analyzed using Cell Quest Pro Software (BD Biosciences, San Jose, CA). Experiments were performed in triplicate in three independent experiments.

### Annexin V assay

The samples were washed with PBS and using Annexin V-FITC and PI staining for determination of phosphatidylserine exposure on the outer plasma membrane. After incubation for 30 min at room temperature protected from light, the samples were quantified by flow cytometry, using a Becton Dickinson FACScan. Experiments were performed in triplicate in three independent experiments.

### Luciferase reporter assays

For luciferase assays, cells (15,000 per well) were plated 24 h before transfection onto white, clear-bottom 96-well plates. The cells were co-transfected with 100 ng of *SP1* 3′-UTR reporter plasmid with 50 nM pre-miR construct using Lipofectamine 2000 (Invitrogen) according to the manufacturer's protocol. Luciferase activity was assayed 24 h after transfection with the Dual-Glo Luciferase Assay System (Promega, Madison, WI) and was measured with an Envision Plate-reader (Perkin Elmer Inc., Wellesley, MA). Samples were tested in triplicate in three independent experiments.

### Western blot analysis

After the indicated treatments, the cells were harvested and proteins prepared using NE-PER Nuclear and Cytoplasmic Extraction Reagent (Pierce, Rockford, IL) with protease inhibitor cocktail (Roche Diagnostics). All extracts were lysed according to the manufacturer's protocol. Total protein content was determined by the Bradford protein method using the BCA protein assay kit (Pierce). Proteins (60 μg) were loaded onto precast 4% stacking, 10% Tris glycine gels and separated by gel electrophoresis followed by electrophoretic blotting onto a PVDF membrane. After washing with Tris-buffered saline, membranes were blocked with 5% BSA/PBS for 1 h. Membranes were incubated with polyclonal rabbit antibodies directed against SP1 and p21 (1∶1000 dilution) (Abcam, UK). The membranes were then incubated with a goat anti-rabbit secondary antibody (1∶1000; Santa Cruz Biotechnology, Santa Cruz, CA), and the blotted proteins were visualized using the enhanced chemi-luminescence detection system (Pierce), with pre-stained markers (Bio-Rad Laboratories, Hercules, CA) as molecular size standards. The intensities of protein bands were quantified using Image J software (National Institutes of Health, Bethesda, MD).

### Statistical analysis

All tests were carried out with the Statistical Package for the Social Sciences software version 17.0 (Chicago, IL, USA). Data are represented as means with standard deviation (SD). Statistical significance was determined with Student t-tests, and *P* values less than 0.05 were considered statistically significant. All statistical tests were two-sided.

## Results

### MiRNA expression profiling in Hela cells induced by IFN-β

**Figure 1:**
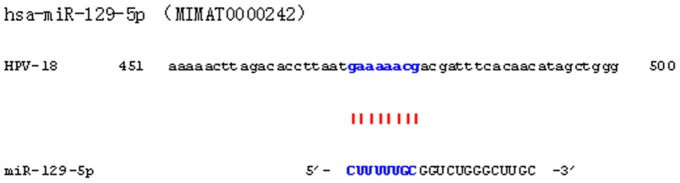
The seed sequence of miR-129-5p displays perfect complementarity with the HPV-18 genome.

**Figure 2:**
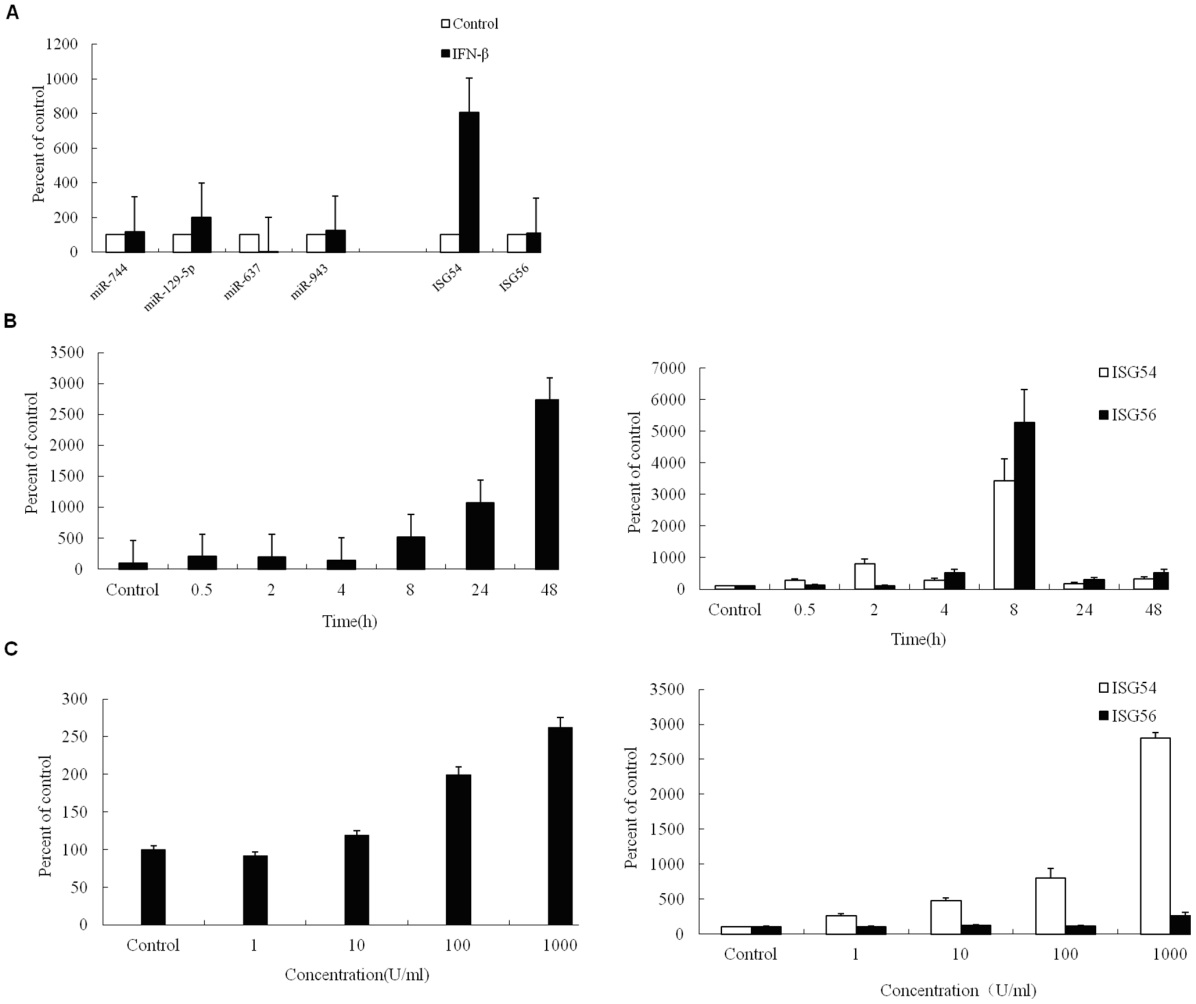
MiR-129-5p can be induced by IFN-β in Hela cells. (A) Validation of the microarray data by qPCR. Triplicate assays were performed for each sample. Inductions of known IFN-regulated genes *ISG54* and *ISG56* are shown for comparsion. (B) Time course of miRNA induction by IFN-β. Hela cells were stimulated with 300 IU ml^−1^ IFN-β for the indicated times, and *miR-129-5p* and *ISG54*/*ISG56* expressions were quantified by qPCR. (C) Dose-response analysis of miRNA induction by IFN-β. Hela cells were stimulated with the indicated doses of IFN-β for 2 h, and *miR-129-5p* and *ISG54*/*ISG56* expressions were quantified by qPCR.

The differentially expressed miRNAs between untreated and IFN-β induced Hela cells were analyzed using microarrays. The expressions of *miR-129-5p*, *miR-637*, *miR-744* and *miR-943* were found to be most highly induced in Hela cells by IFN-β and selected for further analysis (Table 4, and Fig. S1). The seed sequence of *miR-129-5p* displayed perfect complementarity with the HPV-18 genome (Fig. 1). Quantitative real-time PCR (qPCR) was then carried out to validate the microarray analysis, and the results demonstrated that expressions of *miRNA-744*, *miR-943* and *miR-129-5p* were basically consistent with that of the microarray; Time course analysis of expression revealed that induction of *miR-129-5p* occurred rapidly, peaking within 48 h. Similar to the induction of *ISG54*, a well-characterized IFN-β regulated gene, upregulation of *miR-129-5p* followed a classical dose-response curve between 3 and 3000 IU^−1^ ml of IFN-β (Fig. 2).

**Table 4:** Differentially expressed miRNA in Hela cells by IFN-β treatment.

	Name	ID	Fold change	*P* value
Upregulated miRNAs	hsa-miR-744	27568	1.96	0.03
	hsa-miR-129-5p	42467	1.83	0.03
	hsa-miR-637	17354	1.64	0.07
	hsa-miR-943	42696	1.56	0.12
	hsa-miR-2223	11024	1.46	0.13
Downregulated miRNAs	hsa-miR-1279	45775	0.66	0.03
	hsa-miR-526b*	46758	0.69	0.03
	hsa-miR-203	11004	0.64	0.07
	hsa-miR-2295p	11038	0.50	0.09

### *MiR-129-5p* levels gradually decrease with the development of cervical intraepithelial lesions and correlate with HPV-18 E6 and E7 expression

**Figure 3:**
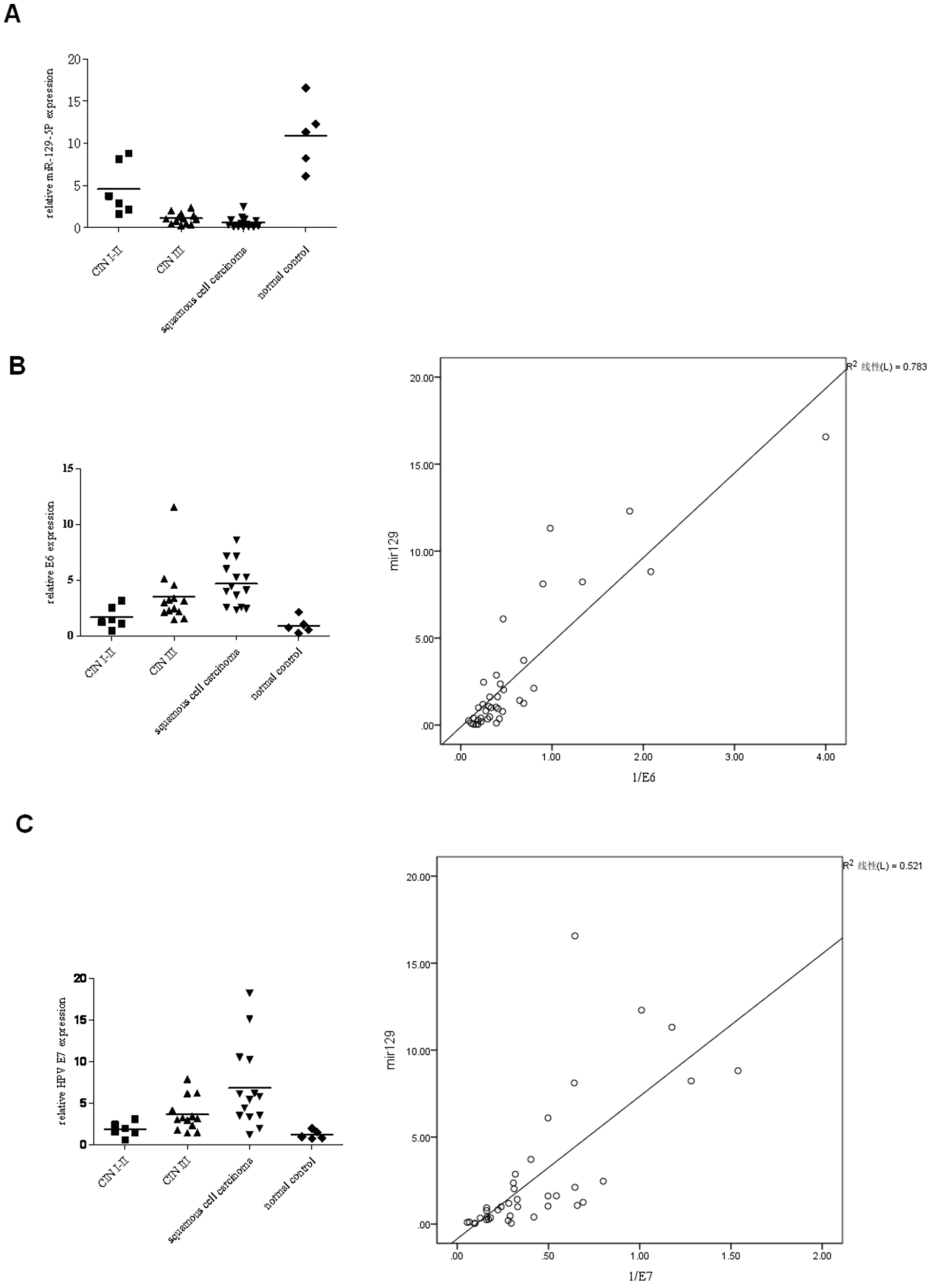
The expression of miRNA-129-5p and HPV-18 E6/E7 in human cervical intraepithelial lesion samples and normal cervical tissues. (A) The expression of miRNA-129-5p in human cervical intraepithelial lesion samples and normal cervical tissues. (B) The expression of HPV-18 E6 in the same samples. (C) The expression of HPV-18 E7 in these samples. (D, E) Correlation between the expressions of HPV-18 E6/E7 and miR-129 in these samples.

By qPCR analysis in human cervical intraepithelial lesion samples and normal cervical tissues, the *miR-129-5p* expression levels were much lower in cancer than in CIN I-II and normal cervical samples (Fig. 3 A). the average expressions of *miR-129-5p* were 10.90±4.01, 4.54±3.12, 1.07±0.65 and 0.57±0.67, respectively, in the normal cervical tissues (control), CIN I-II group, CIN III group, and squamous cell carcinoma groups. Statistically significant differences in the *miR-129-5p* expressions were found when comparing the four groups (*P*<0.05). The clinical stages and pathological grades of the CIN I-II group compared to the other groups were confirmed to be significantly different (*P*<0.05). HPV-18 E6 and E7 expression were significant increased from the normal cervix group, CIN I-II group, CIN III group, and squamous cell carcinoma group (P<0.05, Fig. 3 B, C). The average expressions of HPV-18 E6 were 1.67±0.41, 3.55±0.73, 4.70±0.53 and 0.94±0.33, respectively, in the normal cervical tissues (control), CIN I-II group, CIN III group, and squamous cell carcinoma groups, while the average expressions of HPV-16 E7 were 1.90±0.35, 3.67±0.55, 6.87±1.33 and 1.23±0.24 in these samples. Negative correlation has been found between the expression of miR-129-5p and HPV-18 E6/E7 in cervical tissue samples (P<0.05, Fig. 3 D, E).

### Manipulation of *miR-129-5p* expression in Hela cells modulates HPV-18 *E6* and *E7* viral gene expression

**Figure 4:**
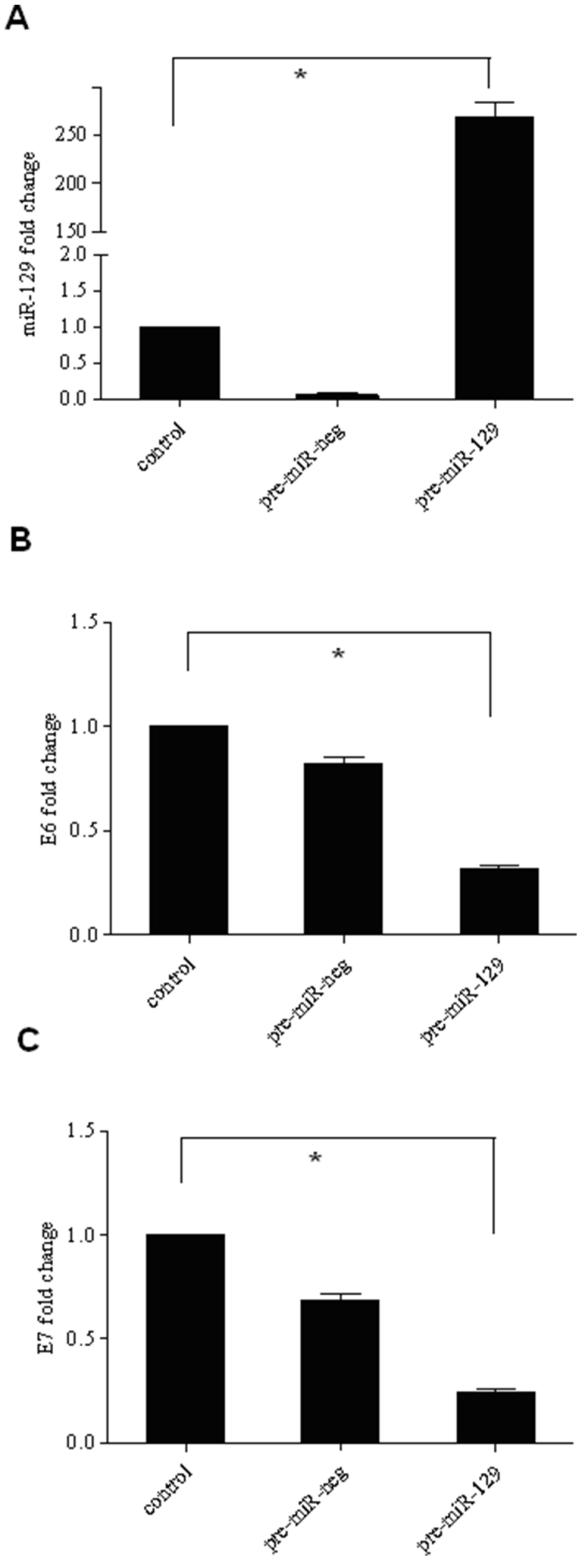
Increase of *miR-129-5p* expression in Hela cells decreased HPV-18 *E6* and *E7* viral gene expressions. Fig.(A) showed the efficiency of miR-129 transfection. Fold changes of the expressions of (B) HPV-18 *E6* and (C) HPV-18 *E7* in Hela cells determined by qPCR. The experiment was repeated twice, each with three replicates. Means (bars) and SDc (error bars) are shown. *P<0.001.

To assess whether *miR-129-5p* has a functional role in the down-regulation of HPV18 *E6* and *E7*, we manipulated the expression level of *miR-129-5p* in Hela cells. The three groups included control cells (untreated), cells transfected with non-specific miRNA control *(pre-miR-negative)*, cells transfected with *pre-miR-129*. At 72 h after transient transfection of pre-miRNAs, the mRNA expressions of HPV-18 *E6* and *E7* in Hela cells were detected by qPCR. Transfection of *pre-miR-129-5p* significantly decreased HPV-18 *E6* and *E7* mRNAs (Fig. 4B, Fig. 4C and Fig. S2), and this effect was highly specific, as *pre-miR-negative* transfection showed no effect on HPV-18 E6 and E7 expression.

### Exogenous *miR-129-5p* inhibits cell proliferation in Hela cells

**Figure 5:**
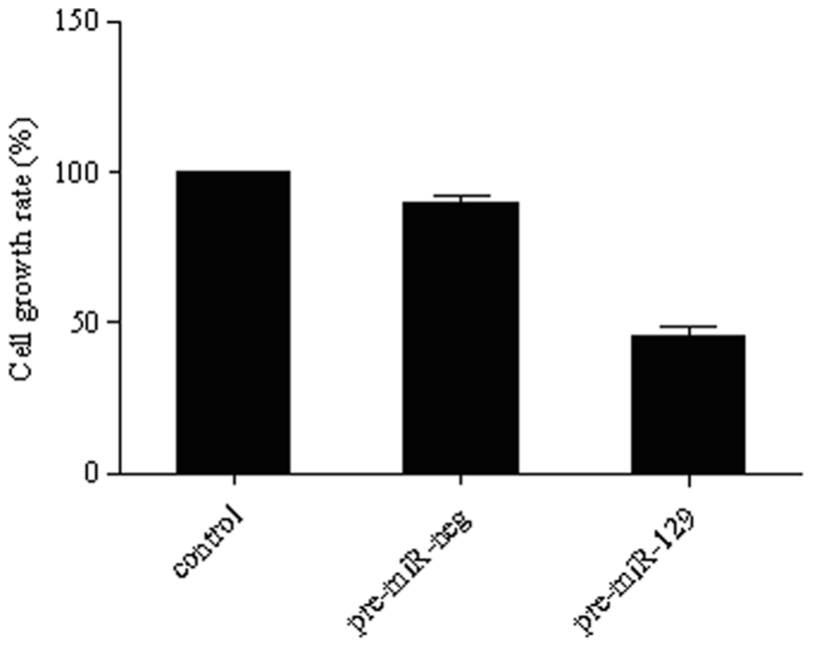
Exogenous miR-129-5p inhibited cell proliferation and promoted apoptosis of Hela cells. Cell growth was measured by an MTT assay. The experiment was repeated at least twice, each with three replicates, and the data are expressed as mean absorbances with SD. *P<0.001; #*P*<0.05.

We next tested whether increased levels of *miR-129-5p* could decrease the proliferative potential of Hela cells as reported in bladder cancer cells. An MTT-based assay was performed to assess the proliferation of Hela cells for up to 72 h after transfection with *miR-129-5p*. The proliferation assay revealed that *miR-129-5p* over-expression inhibited cell growth (Fig. 5), whereas cells transfected with control miRNA continued to grow during this period.

### Exogenous *miR-129-5p* promotes apoptosis and blocks cell cycle progression in Hela cells

**Figure 6:**
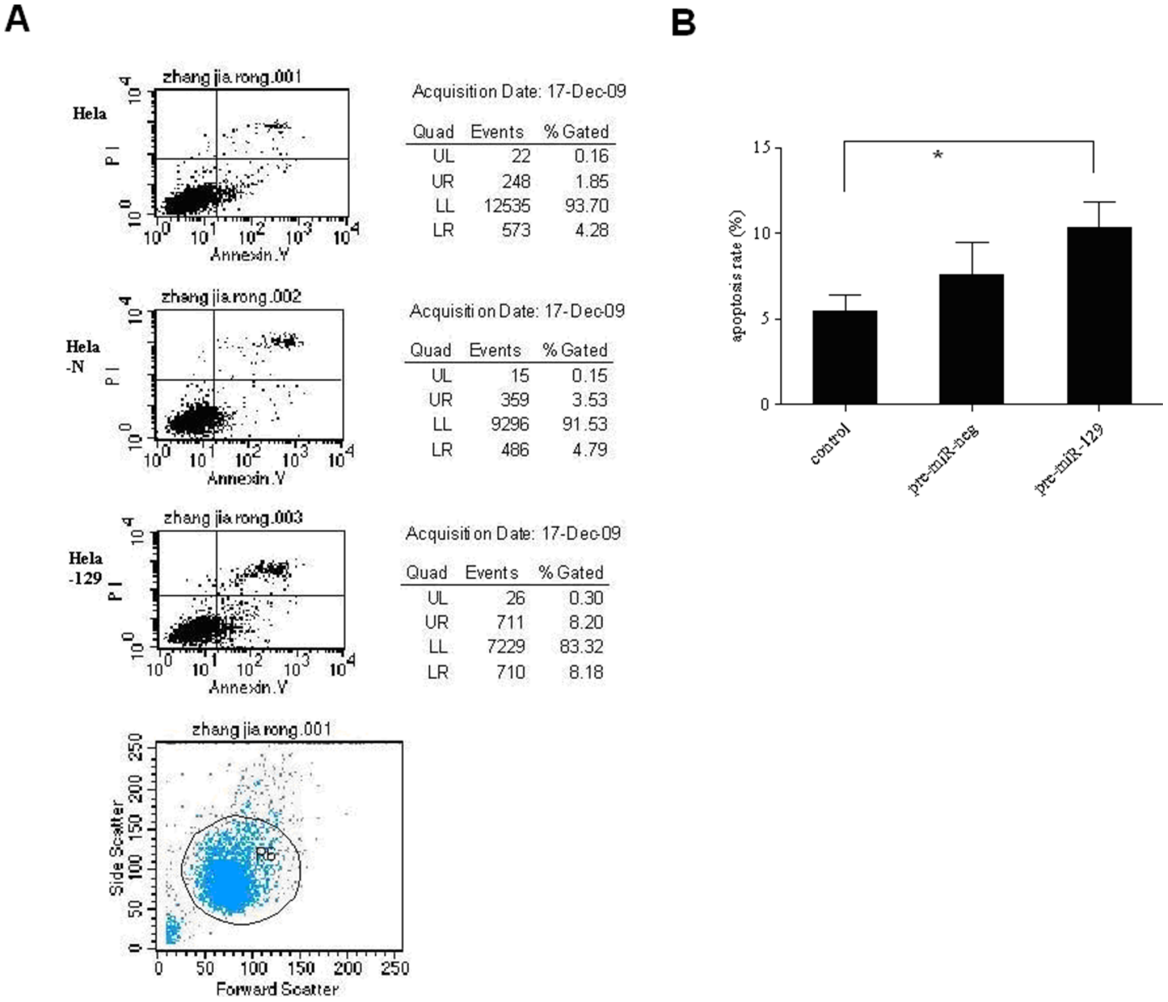
Exogenous *miR-129-5p* promoted apoptosis of Hela cells. Annexin V analysis showed that Hela cells transfected with *pre-miR-129-5p* displayed a significantly greater apoptosis level than the control groups. *P<0.01, #*P*<0.05.

As determined by Annexin V labeling, untreated cells and those treated with *pre-miR-negative* controls remained viable and were primarily Annexin V and propidium iodide (PI) negative. A rightward shift in the fluorescence activated cell sorting (FACS) profile, indicative of apoptosis, was observed for Hela cells transfected with *pre-miR-129-5p* (Fig. 6).

Cell cycle analysis by flow cytometry demonstrated that proportions of cells in G0/G1 and S phases in the negative controls were unchanged compared to untreated cells. Transfection of Hela cells with *pre-miR-129-5p* resulted in an accumulation of cells in the G0/G1 phase and a decrease of cells in the S phase compared with control cells. Flow cytometric analysis suggested that *miR-129-5p* could arrest Hela cells at the G1/S checkpoint and delay the cell cycle from entering into the S phase. Consequently, *miR-129-5p* over-expression slowed down the cell cycle progression and caused a cell cycle G1 phase block, although the difference was not statistically significant.

### *SP1* is a direct target of *miR-129-5p* in Hela cells

**Figure 7:**
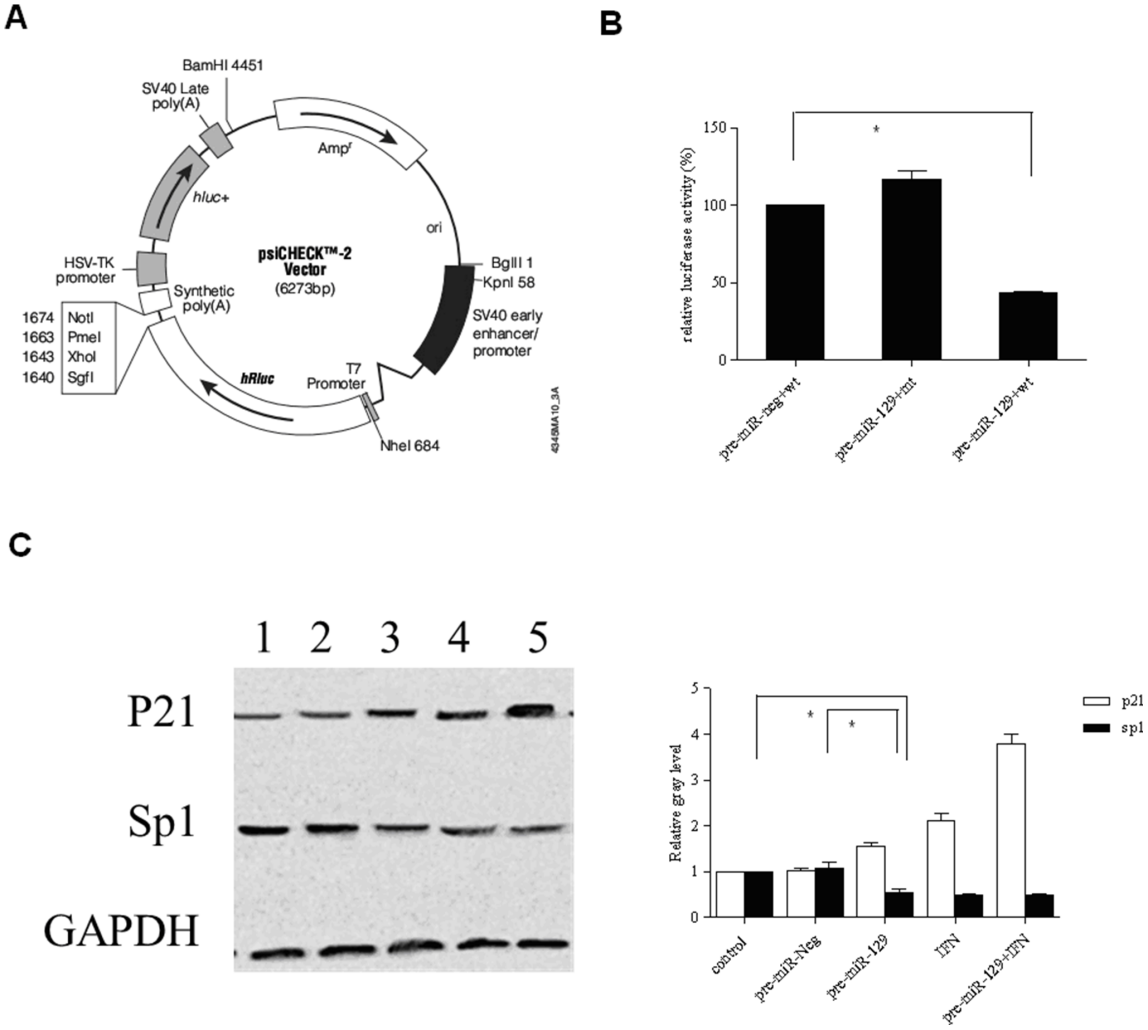
Negative regulation of SP1 by miR-129-5p in Hela cells. (A) Vector restriction map. (B) SP1 was targeted by miR-129-5p via its 3′UTR in Hela cells as assessed using a luciferase assay. (C) Western blot analyses of p21, SP1 and GAPDH in Hela cells after miR-129-5p over-expression. Means (bars) and SD (error bars) are shown.*P<0.05.

A dual-luciferase reporter assay was used to determine whether *miR-129-5p* could bind to the *miR-129-5p* target sites within the *SP1* 3′-UTR. We used constructs containing the predicted seed sequence to test the inhibitory effect of *miR-129-5p*. Transfection of *pre-miR-129-5p* in Hela cells for 48 h decreased luciferase activity as compared with the negative control. While no change in activity was observed when transfected with the control (Fig. 7B), Western blot analysis verified that exogenous *miR-129-5p* could reduce the protein levels of SP1 (Fig. 7C).

## Discussion

In this report, we identified IFN-β induced miRNAs in the HPV-18 positive Hela cells. miRNA microarray analysis showed the expressions of *miR-129-5p*, *miR-637*, *miR-744* and *miR-943* were increased, while miR-526* were downregulated after IFN-β stimulation. *miR-129-5p* was picked out for further study, as bioinformatic analysis revealed that *miR-129-5p* and the HPV-18 viral gene sequences are fully complementary. It has been shown that miR-129-5p was deregulated in several tumor types including endometrial cancer, esophageal cancer, colon cancer and bladder cancer, and its verified target genes included SOX4 [15]–[17], VCP/p97 [18] and Cdk6 [19]. Over-expression of miR-129-5p can inhibit cell growth and induce cell death in bladder cancer [17] and hepatocellular cancer [18], however, its role in cervical cancer is not clear.

We found that miR-129-5p over-expression could inhibit the growth of Hela cells to about 70.23% of control cells. Meanwhile, the transfected pre-miR-129-5p increased the arrest of Hela cells at G0-G1 phase (68.34%), while S phase cells decreased, indicating that cell proliferation was inhibited as DNA synthesis slowed. These results of the effect of miR-129-5p on cell proliferation and cell cycle along with its ability to increase apoptosis were in line with observations in bladder cancer cells [17]. Our study also showed that miR-129-5p expression was induced by IFN-β in a dose and time-dependent manner. These results imply that the anti-proliferation and pro-apoptosis effect of IFN- in cervical cancer may partly due to the induction of miR-129-5p expression.

Cervical cancer caused by HPV infection is mainly associated with the E6 and E7 viral oncogenes. By qPCR analysis, transfection of pre-miR-129-5p in Hela cells was found decrease the expressions of E6 and E7 by 69.01% and 77.15%, respectively. To our knowledge, this is the first report that a IFN-β induced microRNA can down-regulate HPV-18 E6 and E7 viral gene expression. Our further investigation found that the transcription factor SP1 is a direct downstream target of miR-129-5p. SP1 is a sequence-specific DNA binding protein which contains a miR-129-5p binding site in its 3′-UTR. The upstream regulatory regions of HPV-18 genes contain the SP1 binding site, and they have been shown to determine E6 and E7 gene expression [20]–[22]. In the present study, we found that SP1 expression could be down-regulated significantly by over-expressed miR-129-5p. Moreover, we confirmed the existence of a binding site for miR-129-5p at SP1 3′-UTR by luciferase activity assay. Taken together, our results suggest that the induced expression of miR-129-5p by IFN-β suppress the progression of cervical cancer by down-regulating HPV-18 E6 and E7 expression, and SP1 is a direct downstream target of miR-129-5p. An interesting result revealed by our investigation is that miR-129-5p expression was high in CIN I tumors, but it gradually decreased over the course of cancer development and the rise of pathological grade. HPV infection can induce alteration of cellular miRNA expression during the development of cervical cancer. Downregulation of human miR-218 and miR-34a in cervical cancer cells were addressed to the HPV 16 E6 oncogene. An recent study showed that 25 miRNAs were differentially regulated in two or three HPV types (HPV 11/16/45) [23]. It is reasonable that miR-129-5p expression may be suppressed by HPV infection, as the infection rate of high-risk HPV increased through CIN I to cervical cancer. Nevertheless, further study is needed to determine the exact mechanism of decreased miR-129-5p expression during the progression of cervical cancer.

## Supporting Information

Figure S1 **The heat map for the differentially expressed miRNAs between untreated and IFN-β induced Hela cells.**
(TIF)

Figure S2 **A The expression of HPV-18 E6 in hela cells; S2B The expression of HPV-18 E7 in hela cells.**
(TIF)

## References

[1] Bosch FX, Manos MM, Munoz N, Sherman M, Jansen AM, et al (1995) Prevalence of human papillomavirus in cervical cancer: a worldwide perspective. International biological study on cervical cancer (IBSCC) Study Group. J Natl Cancer Inst 87: 796–802. 7791229 10.1093/jnci/87.11.796

[2] Scheffner M, Werness BA, Huibregtse JM, Levine AJ, Howley PM (1990) The E6 oncoprotein encoded by human papillomavirus types 16 and 18 promotes the degradation of p53. Cell 63: 1129–1136. 2175676 10.1016/0092-8674(90)90409-8

[3] Scheffner M, Huibregtse JM, Vierstra RD, Howley PM (1993) The HPV-16 E6 and E6-AP complex functions as a ubiquitin-protein ligase in the ubiquitination of p53. Cell 75: 495–505. 8221889 10.1016/0092-8674(93)90384-3

[4] Werness BA, Levine AJ, Howley PM (1990) Association of human papillomavirus types 16 and 18 E6 proteins with p53. Science 248: 76–79. 2157286 10.1126/science.2157286

[5] Dyson N, Howley PM, Munger K, Harlow E (1989) The human papilloma virus-16 E7 oncoprotein is able to bind to the retinoblastoma gene product. Science 243: 934–937. 2537532 10.1126/science.2537532

[6] Cowland JB, Hother C, Gronbaek K (2007) MicroRNAs and cancer. APMIS 115: 1090–1106. 18042145 10.1111/j.1600-0463.2007.apm_775.xml.x

[7] Li Y, Liu J, Yuan C, Cui B, Zou X, et al (2010) High-risk human papillomavirus reduces the expression of microRNA-218 in women with cervical intraepithelial neoplasia. J Int Med Res 38: 1730–1736. 21309487 10.1177/147323001003800518

[8] Martinez I, Gardiner AS, Board KF, Monzon FA, Edwards RP, et al (2008) Human papillomavirus type 16 reduces the expression of microRNA-218 in cervical carcinoma cells. Oncogene 27: 2575–2582. 17998940 10.1038/sj.onc.1210919 PMC2447163

[9] Wang X, Meyers C, Guo M, Zheng ZM (2011) Upregulation of p18Ink4c expression by oncogenic HPV E6 via p53-miR-34a pathway. Int J Cancer 129: 1362–1372. 21128241 10.1002/ijc.25800 PMC3086996

[10] Yao Q, Xu H, Zhang QQ, Zhou H, Qu LH (2009) MicroRNA-21 promotes cell proliferation and down-regulates the expression of programmed cell death 4 (PDCD4) in HeLa cervical carcinoma cells. Biochem Biophys Res Commun 388: 539–542. 19682430 10.1016/j.bbrc.2009.08.044

[11] Tanaka T, Sugaya S, Kita K, Arai M, Kanda T, et al (2012) Inhibition of cell viability by human IFN-beta is mediated by microRNA-431. Int J Oncol 40: 1470–1476. 22293894 10.3892/ijo.2012.1345

[12] Pedersen IM, Cheng G, Wieland S, Volinia S, Croce CM, et al (2007) Interferon modulation of cellular microRNAs as an antiviral mechanism. Nature 449: 919–922. 17943132 10.1038/nature06205 PMC2748825

[13] Ren C, Cheng X, Lu B, Yang G (2013) Activation of interleukin-6/signal transducer and activator of transcription 3 by human papillomavirus early proteins 6 induces fibroblast senescence to promote cervical tumourigenesis through autocrine and paracrine pathways in tumour microenvironment. Eur J Cancer: in press. 10.1016/j.ejca.2013.07.140 23953057

[14] Qin R, Chen Z, Ding Y, Hao J, Hu J, et al (2013) Long non-coding RNA MEG3 inhibits the proliferation of cervical carcinoma cells through the induction of cell cycle arrest and apoptosis. Neoplasma 60: 486–92. 23790166 10.4149/neo_2013_063

[15] Shen R, Pan S, Qi S, Lin X, Cheng S (2010) Epigenetic repression of microRNA-129-2 leads to overexpression of SOX4 in gastric cancer. Biochem Biophys Res Commun 394: 1047–1052. 20331975 10.1016/j.bbrc.2010.03.121

[16] Huang YW, Liu JC, Deatherage DE, Luo J, Mutch DG, et al (2009) Epigenetic repression of microRNA-129-2 leads to overexpression of SOX4 oncogene in endometrial cancer. Cancer Res 69: 9038–9046. 19887623 10.1158/0008-5472.CAN-09-1499 PMC2789184

[17] Dyrskjot L, Ostenfeld MS, Bramsen JB, Silahtaroglu AN, Lamy P, et al (2009) Genomic profiling of microRNAs in bladder cancer: miR-129 is associated with poor outcome and promotes cell death in vitro. Cancer Res 69: 4851–4860. 19487295 10.1158/0008-5472.CAN-08-4043

[18] Liu Y, Hei Y, Shu Q, Dong J, Gao Y, et al (2012) VCP/p97, Down-Regulated by microRNA-129-5p, Could Regulate the Progression of Hepatocellular Carcinoma. PLoS One 7: e35800. 22536440 10.1371/journal.pone.0035800 PMC3335000

[19] Wu J, Qian J, Li C, Kwok L, Cheng F, et al (2010) miR-129 regulates cell proliferation by downregulating Cdk6 expression. Cell Cycle 9: 1809–1818. 20404570 10.4161/cc.9.9.11535

[20] Hoppe-Seyler F, Butz K (1992) Activation of human papillomavirus type 18 E6-E7 oncogene expression by transcription factor Sp1. Nucleic Acids Res 20: 6701–6706. 1336181 10.1093/nar/20.24.6701 PMC334589

[21] Demeret C, Desaintes C, Yaniv M, Thierry F (1997) Different mechanisms contribute to the E2-mediated transcriptional repression of human papillomavirus type 18 viral oncogenes. J Virol 71: 9343–9349. 9371593 10.1128/jvi.71.12.9343-9349.1997 PMC230237

[22] Demeret C, Le Moal M, Yaniv M, Thierry F (1995) Control of HPV 18 DNA replication by cellular and viral transcription factors. Nucleic Acids Res 23: 4777–4784. 8532518 10.1093/nar/23.23.4777 PMC307464

[23] Dreher A, Rossing M, Kaczkowski B, Andersen DK, Larsen TJ, et al (2011) Differential expression of cellular microRNAs in HPV 11, -16, and -45 transfected cells. Biochem Biophys Res Commun 412: 20–25. 21782796 10.1016/j.bbrc.2011.07.011

