# Supplementary figures and images for: Interferon-β Induced microRNA-129-5p Down-Regulates HPV-18 E6 and E7 Viral Gene Expression by Targeting SP1 in Cervical Cancer Cells
Source: PLoS One. 2013 Dec 16;8(12):e81366. doi: 10.1371/journal.pone.0081366 (PMC3864787; doi:10.1371/journal.pone.0081366)

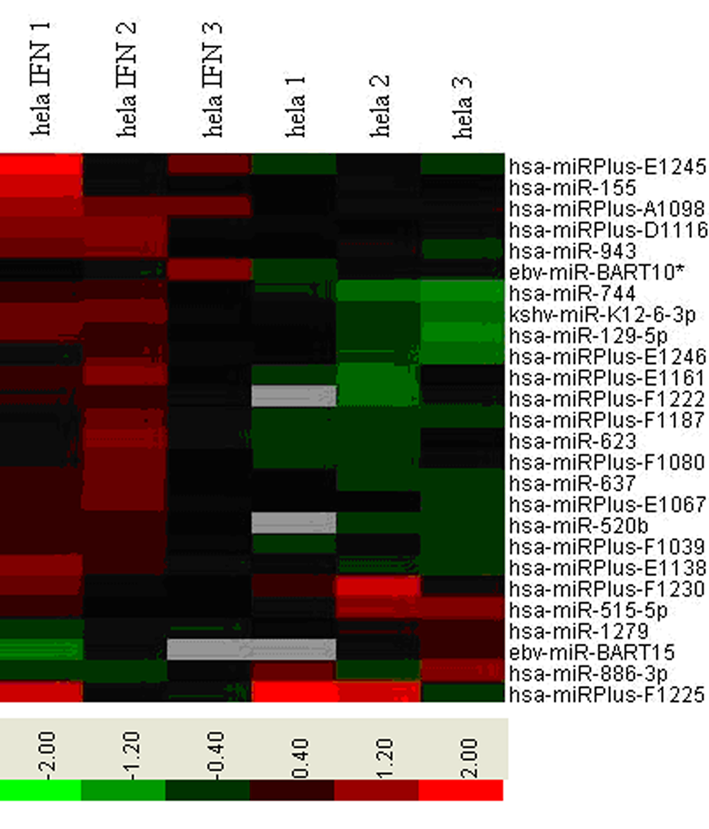

Supplement: Figure S1 — The heat map for the differentially expressed miRNAs between untreated and IFN-β induced Hela cells. (TIF) [file pone.0081366.s001.tif]

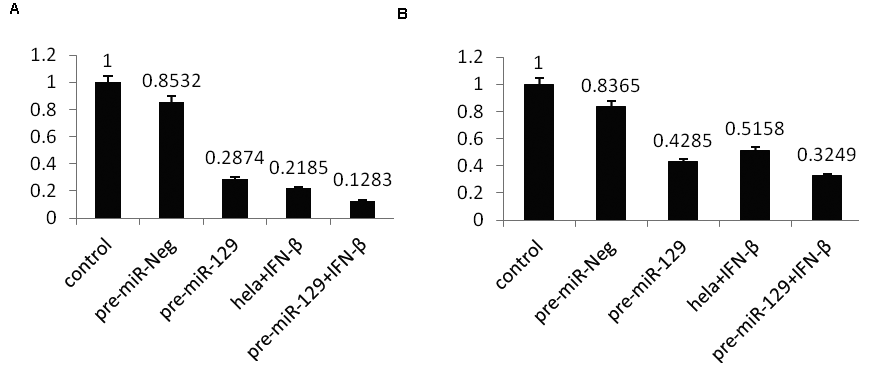

Supplement: Figure S2 — A The expression of HPV-18 E6 in hela cells; S2B The expression of HPV-18 E7 in hela cells. (TIF) [file pone.0081366.s002.tif]
